# Complete Genome Sequence of a *Klebsiella pneumoniae* Strain Carrying *bla*_NDM-1_ on a Multidrug Resistance Plasmid

**DOI:** 10.1128/genomeA.00664-16

**Published:** 2016-07-14

**Authors:** Sean Conlan, Anna F. Lau, NISC Comparative Sequencing Program, Tara N. Palmore, Karen M. Frank, Julia A. Segre

**Affiliations:** a National Human Genome Research Institute, Bethesda, Maryland, USA; b National Institutes of Health Clinical Center, Bethesda, Maryland, USA; c NIH Intramural Sequencing Center, Rockville, Maryland, USA

## Abstract

Here, we report the genome sequence of a *bla*_NDM-1_-positive *Klebsiella pneumoniae* AATZP isolate cultured from a perirectal surveillance swab collected upon admission of a patient to the NIH Clinical Center in 2014. Genome sequencing of this isolate revealed three plasmids, including one carrying the *bla*_NDM-1_ gene encoding resistance to carbapenems.

## GENOME ANNOUNCEMENT

Over the last two decades, there has been a steady and alarming global rise in multidrug-resistant bacteria (1). In particular, plasmid-borne carbapenemase-producing organisms (CPOs) have been cited as an immediate and even “catastrophic” threat to patient health (2, 3). The *Klebsiella pneumoniae* carbapenemase (KPC) enzyme has been the subject of considerable investigation because of its global prevalence (4, 5).

The New Delhi metallo-beta-lactamase (NDM-1), first identified in 2008, has become an important mechanism of carbapenem resistance with worldwide distribution (6). We previously sequenced a *bla*_NDM-1_-positive strain isolated in northeastern Ohio (7) but had never detected *bla*_NDM-1_ in our hospital. In August of 2014, a *Klebsiella pneumoniae* isolate carrying the *bla*_NDM-1_ gene was cultured from a perirectal surveillance swab collected upon admission. The patient came from India and had received extensive treatment in Indian hospitals for a malignancy. We performed whole-genome sequencing to identify whether the *bla*_NDM-1_ gene was plasmid-borne and to enable the development of diagnostic assays for future epidemiological investigations.

Genomic DNA was prepared from an overnight culture, grown on blood agar using the Promega Maxwell 16 nucleic acid purification system (AS1030-tissue kit). Libraries for single-molecule real-time (SMRT) sequencing were constructed using the SMRTbell template kit, version 1.0. The DNA was size-selected for the range of 10 to 50 kb using a BluePippin with a 0.75% gel cassette. Sequencing was performed on the PacBio RSII using P5 polymerase binding and C3 sequencing kits with magnetic bead loading and 180-min acquisition. Genome assemblies were performed using HGAP3 and Quiver as part of SMRT Analysis version 2.3.

*Klebsiella pneumoniae* AATZP belongs to sequence type 147 and has a 5.35-Mb genome and three plasmids. pKPN-04f is 121,030 nucleotides (nt) and is largely syntenic with pKPHS1, a plasmid from *K. pneumoniae* HS11286 (accession no. NC_016838) (8). Two regions of the pKPHS1 sequence are replaced in pKPN-04f, resulting in the loss of a CTX-M-14 extended-spectrum beta-lactamase and replacement of a restriction-modification system. Interestingly, both pKPN-04f and pKPHS1 are predicted to be intact phages by the PHAST tool (9), suggesting a possible phage ancestor. The second plasmid, pKPN-041, is 38,384 nt and carries an aminoglycoside adenylyltransferase and two beta-lactamase genes. Finally, pNDM-1fa shares two large (~20 kb) regions of identity (>99.8%) with plasmid 2 from the northeastern Ohio strain (7). The first region contains three antibiotic resistance genes: *bla*_NDM-1_, a downstream bleomycin resistance gene, and an upstream fluoroquinolone resistance gene. The second region contains plasmid partitioning genes and a restriction-modification system. In plasmid 2, those two regions are separated by a large region containing a *tra* conjugal transfer locus. In pNDM-1fa, that region is replaced with a class 1 integron that is 99.9% identical to one found in plasmids, like pHKU1 (10), and encodes resistance genes for a number of antibiotics, including aminoglycosides, beta-lactams, chloramphenicol, and rifampin. With few published complete *bla*_NDM-1_-positive *Enterobacteriaceae* genomes, these references serve to scaffold short-read assemblies for hospital transmission investigations and studies of plasmid evolution and diversity.

### Nucleotide sequence accession numbers.

This complete genome project has been deposited at DDBJ/EMBL/GenBank under the accession numbers CP014755 to CP014758.
